# Serum Resistin as a Potential Mortality Predictor in Neonatal Sepsis

**DOI:** 10.7759/cureus.55289

**Published:** 2024-02-29

**Authors:** Rashika Jain, Rohan Acharya, Kumud, Kapil Bhalla, Dinkar Yadav, Naman Jain, Sunisha Jakhar

**Affiliations:** 1 Department of Pediatrics, Pandit Bhagwat Dayal Sharma Post Graduate Institute of Medical Sciences (PGIMS), Rohtak, IND

**Keywords:** pediatric disease, resistin, mortality rate in sepsis, morbidity, neonatal sepsis

## Abstract

Aim

The aim of this study was to investigate the utility of serum resistin levels as a prognostic indicator for mortality in neonates diagnosed with sepsis.

Methodology

This one-year prospective study at Pandit Bhagwat Dayal Sharma Post Graduate Institute of Medical Sciences (PGIMS), Rohtak, India, included 151 neonates categorized into two groups based on blood culture results: group 1 (n=86) included those with culture-negative, probable sepsis and group 2 (n=65) included those with culture-positive, proven sepsis. Blood samples obtained pre-treatment underwent comprehensive analysis, including complete blood count, C-reactive protein assessment, micro-erythrocyte sedimentation rate, and resistin level measurement via enzyme-linked immunosorbent assay. The comparison between groups was conducted using either the Student t-test or the Mann-Whitney U test, while correlations were assessed using the Spearman correlation. These analyses were employed to identify the optimal resistin cut-off for distinguishing patients with sepsis. A p-value of <0.05 was considered statistically significant.

Results

This study with 151 neonates diagnosed with sepsis found a significant association (p < 0.05) between elevated serum resistin levels and increased mortality risk. Multivariate analysis confirmed an independent predictive role of resistin. Elevated resistin levels correlate with higher chances of requiring mechanical ventilation and prolonged hospital stays. These findings highlight serum resistin's potential as a prognostic tool for the early identification of high-risk neonatal sepsis patients.

Conclusion

This study highlights the link between elevated serum resistin levels and increased mortality risk in neonatal sepsis, supported by strong multivariate analysis, indicating an independent predictive role. Additionally, resistin correlates with higher chances of mechanical ventilation and prolonged hospitalization, suggesting its potential as a prognostic marker for early identification of high-risk neonatal sepsis cases.

## Introduction

Neonatal sepsis, marked by infection-related indicators within the first month after birth, remains a global healthcare concern despite advancements in maternal and neonatal care [1]. Particularly affecting premature newborns, sepsis significantly contributes to morbidity and mortality. Swift identification of affected infants is crucial for prompt intervention and improved medical management [2].

In the quest for effective prognostic tools to enhance our understanding and management of neonatal sepsis, the role of biomarkers has emerged as a focal point of investigation. Among the myriad of molecules under scrutiny, C-reactive protein (CRP) has long stood as a cornerstone in clinical practice, serving as a reliable marker of inflammation and infection. Its utility in neonatal sepsis, although valuable, has limitations in terms of specificity and early detection, urging the exploration of additional markers that could refine prognostic precision [3,4].

One such candidate biomarker is resistin, a proinflammatory cytokine primarily produced by adipocytes and implicated in the regulation of insulin resistance and glucose metabolism [5]. Although resistin has been studied extensively in the context of adult inflammatory diseases, its role in neonatal sepsis remains a relatively uncharted territory.

Research conducted by Khattab et al. revealed that resistin may be used as a diagnostic tool for newborn sepsis. Additionally, the study found that resistin does not accurately predict death, but it is linked to markers of illness severity [6].

Resistin, recognized for its proinflammatory properties, is proposed as a promising biomarker for sepsis due to its association with systemic inflammation [7]. Initially identified as an adipocytokine hormone in adipose tissue, it was named "resistin" [5] due to its role in inducing insulin resistance in obese mice. In humans, resistin is primarily synthesized by macrophages rather than adipocytes [8]. Despite its unclear involvement in obesity and insulin resistance [9], human resistin plays a crucial role in immune cell recruitment and proinflammatory cytokine release [10]. Linked to inflammatory responses in various disorders [11], interest in using resistin as a sepsis biomarker has surged [12], with limited research focusing on neonates. This study aimed to assess serum resistin's diagnostic potential and prognostic value in neonatal sepsis, a topic previously unexplored.

## Materials and methods

Patients

This prospective study unfolded over the course of one year, spanning from January 2022 to December 2022, and was conducted at Pandit Bhagwat Dayal Sharma Post Graduate Institute of Medical Sciences (PGIMS), Rohtak, India. The study involved collaboration between the Departments of Pediatrics, Neonatology, and Biochemistry. The inclusion criteria comprised neonates exhibiting more than two clinical features of sepsis, along with a positive sepsis screen. Exclusion criteria involved neonates with congenital malformations, genetic syndromes, toxoplasmosis, rubella cytomegalovirus, herpes simplex, HIV (TORCH) infection, surgical conditions, and severe birth asphyxia, and those who received antibiotics before admission. A total of 151 neonates were included using purposive sampling technique, categorized into two groups based on blood culture results: group 1 included 86 neonates with culture-negative, probable sepsis and group 2 included 65 neonates with culture-positive, proven sepsis.

Blood samples

Following the acquisition of a comprehensive medical history, every participant underwent a thorough clinical examination. Before commencing antibiotic treatment, blood samples were obtained to do a comprehensive blood count, measure CRP, assess micro-erythrocyte sedimentation rate (micro-ESR), evaluate resistin levels, and perform blood culture. CRP was assessed using a Latex agglutination assay, considering titers by 1:1 dilution as positive. For resistin evaluation, 1 mL of blood was aseptically collected by venipuncture, left to clot, and then centrifuged for 10 minutes at 5,000 rpm. The separated sera were stored at -20°C until the time of assay. The measurement of resistin was performed using a commercially available sandwich enzyme-linked immunosorbent assay (ELISA) called Resistin Human ELISA (BioVendor Laboratory Medicine, Inc., Brno, Czech Republic). The test was conducted on venous blood samples that had been maintained at a temperature of -20°C until the time of the assay.

Statistical analysis

SPSS Version 25 (IBM Corp., Armonk, NY) was used to perform the statistical tests. The mean and standard deviation were used to describe all continuous values, whereas numbers and percentages were used to represent categorical data. Descriptive statistics were used to determine the frequencies and proportions. The statistics were presented as the mean±standard deviation for continuous variables that followed a normal distribution or as the median (with the minimum and maximum values) for continuous variables that were not normally distributed. Categorical variables were given as proportions. A p-value of <0.05 was deemed to be statistically significant. The groups were compared using the Student t-test. The Spearman correlation test was used to assess the correlations.

## Results

The research included a total of 151 newborns. The neonates were categorized into two groups according to their blood culture results. Group 1 included newborns who likely had sepsis but their cultures tested negative and group 2 included newborns who had confirmed sepsis (with positive culture results).

Table 1 provides a detailed comparison of the demographic profiles between clinically diagnosed sepsis cases (n=86) and those confirmed through culture (n=65). The data revealed that 45.35% of clinically diagnosed cases and 55.38% of culture-proven cases were preterm, with no statistically significant difference (p=0.458). Notably, late preterm and term births showed similar distributions in both groups. The mean gestation period was slightly higher in clinically diagnosed cases (34.61 ± 3.05 weeks) compared to culture-proven cases (33.93 ± 3.12 weeks), but this difference was not significant (p=0.168). Birth weight categories (<1 kg, 1-1.49 kg, 1.5-1.99 kg, 2-2.5 kg, >2.5 kg) exhibited no significant differences between the two groups. Interestingly, while the mean birth weight was slightly higher in clinically diagnosed cases, the difference was not statistically significant (p=0.079). Gender distribution, mode of delivery (vaginal or cesarean), and the administration of steroid cover also showed no significant differences. Onset of sepsis, particularly early onset sepsis (EOS), was significantly higher in culture-proven cases (73.85%) compared to clinically diagnosed cases (55.81%, p=0.023). Other significant differences included the occurrence of preterm premature rupture of membranes (PPROM) (p=0.019), meningitis (p<0.0001), duration of hospital stay (p<0.0001), ventilation methods (p<0.0001), and mortality rates (p=0.001).

**Table 1:** Comparison of demographic profile between clinical and culture-proven sepsis PPROM, preterm premature rupture of membranes; EOS, early onset sepsis; LOS, late-onset sepsis; ANC, absolute neutrophil count

Demographic profile	Clinical (n=86)	Proven (n=65)	Total	P-value
Term/preterm				
Preterm	39 (45.35%)	36 (55.38%)	75 (49.67%)	0.458
Late preterm	18 (20.93%)	12 (18.46%)	30 (19.87%)	
Term	29 (33.72%)	17 (26.15%)	46 (30.46%)	
Period of gestation in weeks				
Mean ± SD	34.61 ± 3.05	33.93 ± 3.12	34.31 ± 3.09	0.168
Median (IQR)	34.75 (32.375-37.225)	33.7 (31.6-37)	34.3 (32.275-37)	
Range	26.8-41.6	28.1-40	26.8-41.6	
Birth weight (kg)				
<1 kg	3 (3.49%)	4 (6.15%)	7 (4.64%)	0.65
1-1.49	24 (27.91%)	24 (36.92%)	48 (31.79%)	
1.5-1.99 kg	26 (30.23%)	15 (23.08%)	41 (27.15%)	
2-2.5 kg	13 (15.12%)	8 (12.31%)	21 (13.91%)	
>2.5 kg	20 (23.26%)	14 (21.54%)	34 (22.52%)	
Mean ± SD	1.89 ± 0.6	1.74 ± 0.6	1.82 ± 0.61	0.079
Median (IQR)	1.72 (1.472-2.38)	1.6 (1.2-2.3)	1.68 (1.325-2.36)	
Range	0.58-3.37	0.88-3	0.58-3.37	
Gender				
Female	35 (40.70%)	27 (41.54%)	62 (41.06%)	0.917
Male	51 (59.30%)	38 (58.46%)	89 (58.94%)	
PPROM				
No	69 (80.23%)	41 (63.08%)	110 (72.85%)	0.019
Yes	17 (19.77%)	24 (36.92%)	41 (27.15%)	
Mode of delivery				
Vaginal	64 (74.42%)	52 (80%)	116 (76.82%)	0.421
Cesarean	22 (25.58%)	13 (20%)	35 (23.18%)	
Steroid cover				
Complete	18 (36.73%)	9 (21.43%)	27 (29.67%)	0.087
Incomplete	19 (38.78%)	26 (61.90%)	45 (49.45%)	
Absent	12 (24.49%)	7 (16.67%)	19 (20.88%)	
Onset of sepsis				
EOS	48 (55.81%)	48 (73.85%)	96 (63.58%)	0.023
LOS	38 (44.19%)	17 (26.15%)	55 (36.42%)	
Total leucocyte count(cells/mm³)				
Negative	61 (70.93%)	36 (55.38%)	97 (64.24%)	0.048
Positive	25 (29.07%)	29 (44.62%)	54 (35.76%)	
Mean ± SD	11,446.51 ± 15,578.29	9,799.08 ± 7,777.73	10,737.35 ± 12,806.27	0.223
Median (IQR)	8,000 (5,000-15,000)	6,000 (3,500-17,000)	6,800 (4,200-15,000)	
Range	1,500-140,000	1,140-32,000	1,140-140,000	
ANC (cells/mm³)				
Negative	69 (80.23%)	42 (64.62%)	111 (73.51%)	0.031
Positive	17 (19.77%)	23 (35.38%)	40 (26.49%)	
Mean ± SD	6,301.76 ± 5,986.85	6,022.03 ± 6,394.54	6,181.34 ± 6,146.25	0.193
Median (IQR)	4,040 (1,794-8,960)	2,438 (1,271-10,500)	2,860 (1,400-9,795)	
Range	520-32,300	650-24,000	520-32,300	
Duration of hospital stay(days)				
<7 days	18 (21.95%)	0 (0%)	18 (13.64%)	0.0001
≥7 days	64 (78.05%)	50 (100%)	114 (86.36%)	
Mean ± SD	11.12 ± 6.44	23.06 ± 7.48	15.64 ± 8.96	<0.0001
Median (IQR)	10 (7-14)	22 (16.5-27.25)	14 (7-21)	
Range	5-40	14-42	5-42	
Meningitis				
No	80 (93.02%)	33 (50.77%)	113 (74.83%)	<0.0001
Yes	6 (6.98%)	32 (49.23%)	38 (25.17%)	
Ventilation				
Non-invasive	72 (93.51%)	36 (57.14%)	108 (77.14%)	<0.0001
Invasive	5 (6.49%)	27 (42.86%)	32 (22.86%)	
Mortality				
No	82 (95.35%)	50 (76.92%)	132 (87.42%)	0.001
Yes	4 (4.65%)	15 (23.08%)	19 (12.58%)	

**Figure 1:**
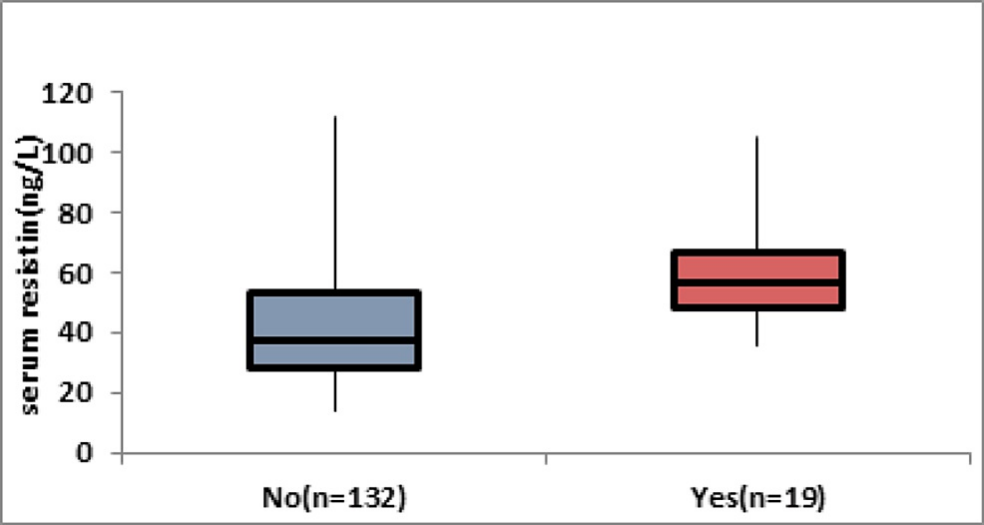
Correlation between serum resistin levels (measured in ng/L) and mortality Image Credit: Dr. Rashika Jain

Table 2 and Figure 1 show the correlation between serum resistin levels (measured in ng/L) and mortality in the population under investigation. The data are divided into two groups: "No" for individuals who did not experience mortality (n=132) and "Yes" for those who did (n=19). The mean serum resistin level in the group with mortality (59.08 ± 17.84 ng/L) is significantly higher than in the group without mortality (40.57 ± 16.84 ng/L), as indicated by the low p-value (<0.0001). Similarly, the median values and interquartile ranges (IQRs) for both groups further emphasize this difference, with the group experiencing mortality showing higher values. The range of serum resistin levels is also wider in the mortality group (35.4-105 ng/L) compared to the non-mortality group (14-112 ng/L).

**Table 2:** Association between serum resistin (ng/L) and mortality Note: p-value of<0.0001 denotes statistical significance

Serum resistin (ng/L)	No (n=132)	Yes (n=19)	Total	P-value
Mean ± SD	40.57 ± 16.84	59.08 ± 17.84	42.9 ± 17.99	<0.0001
Median (IQR)	37 (27.95-52.9)	56.2 (48.25-66.45)	39.8 (28.65-54.65)	
Range	14-112	35.4-105	14-112	

**Figure 2:**
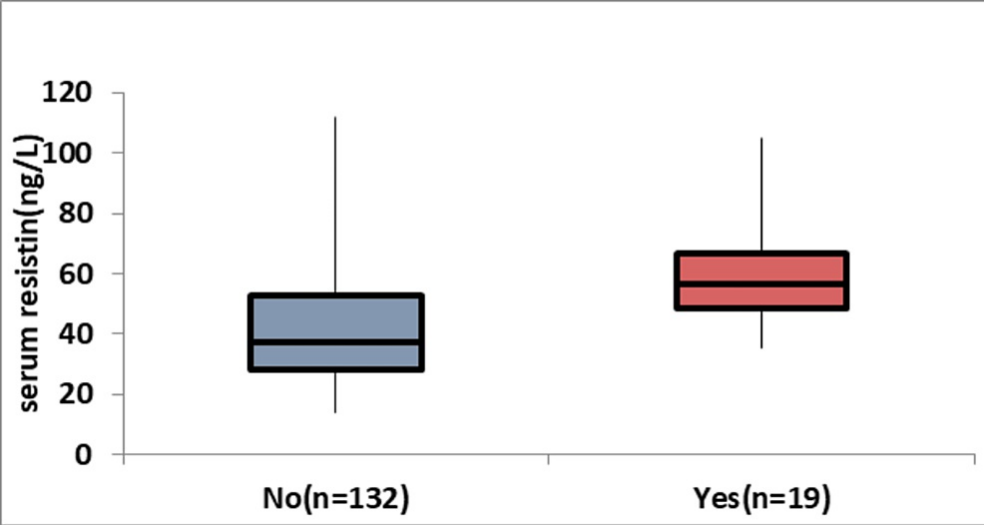
Association between serum resistin (ng/L) and ventilation Image Credit: Dr. Rohan Acharya

Table 3 and Figure 2 show the correlation between serum resistin levels (ng/L) and the type of ventilation (non-invasive or invasive) in a given population. The results indicate a significant difference in mean serum resistin levels between non-invasive (39.95 ± 16.68 ng/L) and invasive ventilation groups (56.95 ± 15.15 ng/L), with a p-value of <0.0001. The median values and IQRs further support this difference, with the invasive ventilation group exhibiting higher serum resistin levels. The wider range in the invasive ventilation group suggests greater variability in serum resistin levels in patients requiring more intensive respiratory support.

**Table 3:** Association between serum resistin (ng/L) and ventilation Note: p-value of<0.0001 denotes statistical significance

Serum resistin (ng/L)	Non-invasive (n=108)	Invasive (n=32)	Total	P-value
Mean ± SD	39.95 ± 16.68	56.95 ± 15.15	43.84 ± 17.8	<0.0001
Median (IQR)	36.6	56	40.85	
	(28-49.7)	(51.8-62.75)	(29-54.85)	
Range	15.5-112	23.8-105	15.5-112	

**Figure 3:**
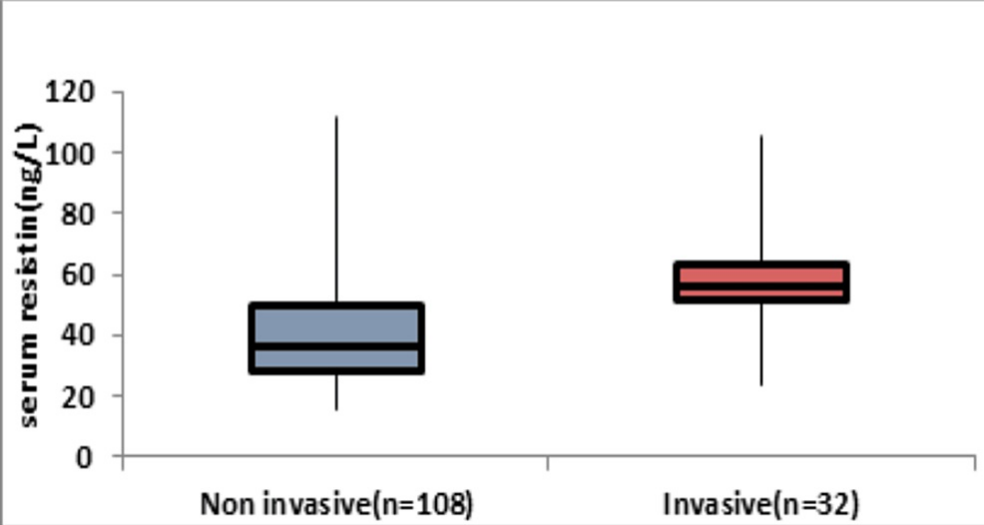
Association between serum resistin (ng/L) and duration of hospital stay (days). Image Credit: Dr. Kumud

Table 4 and Figure 3 show the correlation between serum resistin levels (ng/L) and the duration of hospital stay (categorized as <7 days and ≥7 days) in a given population. The results reveal a substantial disparity in mean serum resistin levels among the two duration groups, with those staying for <7 days having a significantly lower mean (21.01 ± 3.93 ng/L) compared to those with a longer stay (43.66 ± 15.99 ng/L), with a p-value of <0.0001. The median values and IQR further confirm this distinction, indicating higher serum resistin levels in the group with a hospital stay of ≥7 days. The wider range in the group with a longer stay suggests greater variability in serum resistin levels, possibly signifying a relationship between elevated resistin levels and an extended duration of hospitalization.

**Table 4:** Correlation between serum resistin levels (measured in ng/L) and duration of hospital stay (days).

Serum resistin (ng/L)	<7 days (n=18)	≥7 days (n=114)	Total	P-value
Mean ± SD	21.01 ± 3.93	43.66 ± 15.99	40.57 ± 16.84	<0.0001
Median (IQR)	20.55 (18.35-23.85)	40.85 (31.625-53.575)	37 (27.95-52.9)	
Range	14-28.8	15.5-112	14-112	

## Discussion

The presence of neonatal sepsis is a significant problem in pediatric healthcare, requiring precise predictive techniques to enable appropriate management. Serum resistin, an adipokine linked to inflammation, has lately gained interest for its ability to predict death in newborn sepsis [13]. This research investigates the correlation between serum resistin levels and negative outcomes, with the goal of providing significant knowledge in the area of predicting newborn sepsis. Given the ongoing worry over mortality in sepsis therapy, comprehending the predictive capacities of serum resistin might provide doctors with a beneficial instrument for assessing risk and making educated decisions in newborn care.

The present study compares demographic profiles between clinically diagnosed neonatal sepsis cases (n=86) and those confirmed through culture (n=65). Analysis reveals that preterm births accounted for 45.35% in clinically diagnosed cases and 55.38% in culture-proven cases, exhibiting no statistically significant difference (p=0.458). Late preterm and term births displayed similar distributions in both groups. While clinically diagnosed cases showed a slightly higher mean gestation period (34.61 ± 3.05 weeks) compared to culture-proven cases (33.93 ± 3.12 weeks), this difference was not significant (p=0.168). Birth weight categories (<1 kg, 1-1.49 kg, 1.5-1.99 kg, 2-2.5 kg, >2.5 kg) demonstrated no significant differences. Although clinically diagnosed cases had a slightly higher mean birth weight, the distinction was not statistically significant (p=0.079). Gender distribution, mode of delivery, and steroid cover administration displayed no significant differences. EOS was notably higher in culture-proven cases (73.85%) compared to clinically diagnosed cases (55.81%, p=0.023). Additionally, significant differences were observed in PPROM occurrence (p=0.019), meningitis (p<0.0001), duration of hospital stay (p<0.0001), ventilation methods (p<0.0001), and mortality rates (p=0.001).

Unlike the results presented in our research, Khattab et al. provided a concise comparison between the control group (n=30) and the sick group (n=60), highlighting clear disparities. The patient group, with a median admission age of 9.5 days, did not show a statistically significant difference when contrasted with the control group (8 days, p=0.56). Surprisingly, the group of individuals who were unwell had a significantly greater percentage of females (51.7%) in comparison to the control group (33.3%, p<0.001). The patient group exhibited a lower entry weight (2.41 ± 0.62 kg, p=0.009) and a somewhat earlier gestational age (37 weeks) in comparison to the control group (38 weeks, p=0.016). Distinctive features such as cesarean delivery, severe sepsis, duration of stay in the neonatal intensive care unit, breathing techniques, and death rates were only seen in the group of patients. Neonates with sepsis may be separated from the control group by seeing elevated levels of CRP, platelets, absolute neutrophil count, immature to total neutrophil ratio, neutrophil-to-lymphocyte ratio, and red cell distribution width, and especially higher resistin levels (all p<0.001). These characteristics are of great value in differentiating between the two groups [6].

In this study, serum resistin levels (ng/L) were investigated for their association with mortality in the studied population. The mean resistin level in the mortality group (59.08 ± 17.84 ng/L) significantly exceeded that in the non-mortality group (40.57 ± 16.84 ng/L) with a low p-value (<0.0001). Consistently, median values and IQRs emphasized this distinction, highlighting elevated resistin levels in the mortality group. The wider range of resistin levels in the mortality group (35.4-105 ng/L) compared to the non-mortality group (14-112 ng/L) underscores the potential of serum resistin as a robust prognostic marker for mortality in this population.

Unlike the results shown in our investigation, Sunden-Cullberg et al. performed a study that included 29 patients with severe sepsis and 66 patients with septic shock, using septic shock as an indicator of death. The research demonstrated a significant elevation in resistin levels in comparison to the control group, indicating associations with initial Acute Physiological Assessment Chronic Health Evaluation (APACHE II) scores and daily Sequential Organ Failure Assessment (SOFA) scores at various time intervals. Although there was an increase in resistin levels among non-survivors, the difference did not achieve statistical significance. Gram-positive sepsis showed somewhat higher resistin levels in comparison to gram-negative sepsis. The research underlined the extended increase in serum resistin levels compared to other cytokines, underscoring its function as an acute-phase protein in sepsis and septic shock. Resistin had a distinct connection with the severity of the illness, as shown by APACHE II and SOFA scores, and showed a correlation with well-established indicators of cytokines, such as IL-6, IL-8, IL-10, TNF-α, and sepsis [14].

In our study, we explore the link between serum resistin levels (ng/L) and ventilation mode (non-invasive or invasive) in a specific population. The data expose a notable contrast in mean serum resistin levels, with non-invasive ventilation (39.95 ± 16.68 ng/L) differing significantly from invasive ventilation (56.95 ± 15.15 ng/L) (p<0.0001). Median values and IQRs confirm this distinction, emphasizing higher serum resistin levels in the invasive ventilation group, indicative of increased variability.

Our study's results differ from those of Saboktakin et al. [15], who found elevated resistin levels in patients staying in the pediatric intensive care unit (PICU), especially those receiving mechanical ventilation. This aligns with prior research findings [16-18]. These findings emphasize sepsis as a crucial element in acute respiratory distress syndrome since sepsis enhances vulnerability to ventilator-induced lung damage [19,20]. The intricate interaction among sepsis, length of stay in the PICU, and mechanical ventilation suggests a reciprocal influence. The research specifically targeted pediatric patients diagnosed with sepsis, ranging from 1 month to 12 years of age. The findings indicated that utilizing a threshold of 5.2 ng/mL on the first day of examination exhibited a notable degree of sensitivity (0.824) and specificity (0.72). The dependability of the determined threshold was emphasized by the use of consistent cut-off points for several age groups, ranging from 1 month to 2 years and 25 months to 12 years. Significantly, case-control B had a higher threshold value compared to case-control A, suggesting differences in resistin levels across the case groups [15].

In our current study, we investigated the correlation between serum resistin levels (ng/L) and the duration of hospital stay in a specific population. The results revealed a significant difference in mean serum resistin levels between those with a hospital stay of <7 days (21.01 ± 3.93 ng/L) and those with a longer stay (43.66 ± 15.99 ng/L), supported by a highly significant p-value (p<0.0001). Median values and IQRs further confirmed higher serum resistin levels in the group with a hospital stay of ≥7 days. Similarly, neonates who had a longer period of hospital stay (≥7 days) had a significantly greater average value of resistin in comparison to those with a shorter duration of hospital stay (<7 days), with a p-value of less than 0.0001 [6].

The study investigating serum resistin in neonatal sepsis is subject to certain limitations. Firstly, its one-year prospective design spanning from January 2022 to December 2022 may be susceptible to variations due to seasonal changes and evolving healthcare practices. Additionally, the reliance on clinical features and a positive sepsis screen for inclusion criteria introduce subjectivity into the study population selection process. While exclusion criteria are necessary, they may inadvertently restrict the generalizability of the findings. Furthermore, categorizing neonates based solely on blood culture results has inherent limitations owing to the sensitivity and specificity of blood cultures. Moreover, the role of resistin in neonatal sepsis remains relatively unexplored, and the study's exclusive focus on resistin overlooks the potential contributions of other biomarkers. Lastly, the sample size of 151 neonates may limit the statistical power of the study, highlighting the need for larger cohorts to ensure robust findings.

## Conclusions

In our study, we focused on the realm of neonatal sepsis, where timely and precise clinical decisions were crucial. This prospective study uncovered a promising avenue for prognostication. Serum resistin, identified as a robust biomarker, demonstrated a clear association with adverse outcomes in neonatal sepsis, offering enhanced predictive capabilities. Elevated resistin levels signifying increased inflammation emerged as a valuable tool for risk stratification, enabling healthcare practitioners to make better-informed choices about patient care. The potential optimization of resource allocation and improved efficiency in neonatal care further underscore the clinical significance of our findings. Despite these promising results, the study acknowledges the need for larger, more diverse cohorts to validate and refine the predictive utility of resistin. Continued research, particularly into the molecular mechanisms underlying resistin-CRP synergy, presents an exciting avenue for future investigation.
